# The synergistic interaction between the calcineurin B subunit and IFN-*γ* enhances macrophage antitumor activity

**DOI:** 10.1038/cddis.2015.92

**Published:** 2015-05-07

**Authors:** Z Su, R Yang, W Zhang, L Xu, Y Zhong, Y Yin, J Cen, J P DeWitt, Q Wei

**Affiliations:** 1 Department of Biochemistry and Molecular Biology, Beijing Normal University, Gene Engineering and Biotechnology Beijing Key Laboratory, Beijing, PR China; 2 Department of Biochemistry and Molecular Biology, Medical School, Southeast University, Nanjing, Jiangsu, PR China; 3 Department of Cell Biology, Harvard Medical School, Boston, MA 02115, USA

## Abstract

Macrophages are involved in tumor growth and progression. They infiltrate into tumors and cause inflammation, which creates a microenvironment favoring tumor growth and metastasis. However, certain stimuli may induce macrophages to act as tumor terminators. Here we report that the calcineurin B subunit (CnB) synergizes with IFN-*γ* to make macrophages highly cytotoxic to cancer cells. Furthermore, CnB and IFN-*γ* act synergistically to polarize mouse tumor-associated macrophages, as well as human monocyte-derived macrophages to an M1-like phenotype. This synergy is mediated by the crosstalk between CnB-engaged integrin *α*M-p38 MAPK signaling and IFN-*γ*-initiated p38/PKC-*δ*/Jak2 signaling. Interestingly, the signal transducer and activator of transcription 1 (STAT1) is a key factor that orchestrates the synergy of CnB and IFN-*γ*, and the phosphorylation status at Ser727 and Tyr701 of STAT1 is directly regulated by CnB and IFN-*γ*.

Calcineurin (Cn) is the only known Ca^2+^/calmodulin-dependent serine/threonine protein phosphatase; it is a heterodimer composed of a 61-kDa catalytic subunit (CnA) and a 19-kDa regulatory subunit (CnB).^1, 2^ This enzyme, expressed in various cell types, can modulate T-cell activation,^3^ neuronal excitability,^4, 5^ apoptosis,^6, 7^ cardiac hypertrophy,^8, 9^ and inflammation.^10^

Although CnB can regulate the phosphatase activity of the CnA,^11, 12^ this subunit also has additional non-regulatory roles. Cytosolic CnB can interact with tubulin, heat shock protein 60,^13^ procaspase-3,^14^ and PSMA7^15^ and may therefore function in modulating apoptosis, as well as the ubiquitin/proteasome pathway. CnB also induces dendritic cell maturation and activation and enhances antigen presentation. In fact, this function of CnB has been utilized as the basis for a novel adjuvant for both cancer vaccine^16^ and the Engerix-B HBV vaccine.^17^ Additionally, we have reported that CnB can also interact with integrin *α*M and induce TNF-related apoptosis-inducing ligand (TRAIL) expression in macrophages.^18^^,19^ Furthermore, intraperitoneal injection of CnB protein can prolong the survival of mice bearing H22 ascitic tumors and inhibit the growth of S180 sarcomas in mice.^20^

IFN-*γ*, the sole type II interferon, is a cytokine that is critical for both innate and adaptive immunity to viral and bacterial infections and for tumor surveillance.^21, 22, 23, 24^ It is mainly produced by CD4+ T helper cells, CD8+ cytotoxic T lymphocytes, and NK cells.^21, 25, 26^ It is also produced by B cells, NKT cells, antigen-presenting cells such as monocytes/macrophages, and dendritic cells.^27, 28^ The cellular response to interferons is mainly mediated through signal transducer and activator of transcription 1 (STAT1).^29^ STAT1 contains two major phosphorylation sites: Tyr701 and Ser727; IFN-*γ*-induced STAT1 Tyr701 phosphorylation is thought to be mediated by Janus kinase 2 (Jak2), while Ser727 phosphorylation is thought to be mediated by p38 mitogen-activated protein kinase (MAPK) or protein kinase C-*δ* (PKC-*δ*).^30, 31, 32^ Phosphorylation at Tyr701 is thought to be important for the formation of a homodimer (i.e., STAT1:STAT1) and heterotrimers (i.e., STAT1:STAT1:interferon regulatory factor (IRF)-9 and STAT1:STAT2:IRF-9), which translocate to the nucleus, bind to IFN-*γ* response elements, and initiate transcription of a series of IFN-*γ*-regulated genes such as ICAM-1, CXC-chemokine ligand (CXCL) 9,10, IRF-1, 2, TRAIL, inducible nitric oxide synthase (iNOS), and IFN-*β*. In contrast to the role of phosphorylation at Tyr701, phosphorylation of STAT1 at Ser727 is required for maximal transcription of target genes,^30, 33, 34^ most likely by promoting interaction with proteins such as MCM-5 and BRCA1.^35, 36^ Cellular stress (e.g., UV irradiation), cytokines (e.g., IL-2, IL-12), and TLR agonists (e.g., LPS) do not induce STAT1 Tyr701 phosphorylation but stimulate STAT1 Ser727 phosphorylation.^31, 37, 38, 39^ Thus phosphorylation of STAT on Ser727 functions as a key target linking extracellular stimuli with IFN-*γ* signaling, which in turn leads to maximal activation of innate immune cells.^31, 38, 39^

Tumors have a complex cellular program that supports both their growth and expansion. In the micro-ecosystem, innate immune cells, especially macrophages cause inflammation and serve a critical role in tumor progression by supporting angiogenesis, promoting tumor cell invasion, migration, and intravasation, as well as suppressing antitumor immune responses.^40, 41^ Usually, macrophages are divided into two categories based on their secretory profile and function: M1-like subtype and M2-like subtype. M1-like branches are classically activated macrophages in response to IFN-*γ* with Toll-like receptor engagement and are characterized by their cytotoxity and by high expression of IL-12, CD86, CXCL9, CXCL10, IL-23, IL-6, and TRAIL.^42, 43^ In contrast, M2-like branches are alternatively activated macrophages in response to IL-4 and IL-13 and are characterized by high expression of CD206, arginase-1 (ARG1), and IL-10.^44^ In general, tumor-associated macrophages (TAMs) exhibit M2-like properties and promote tumor progression. However, the precise ratio of M1 to M2 macrophages in the tumor and its microenvironment may determine the tumor viability and progression. In nonprogressing or regressing tumors, macrophages are shifted to an M1-like subtype; in malignant tumors, macrophages resemble an M2-like subtype.^40^ Specific microenvironmental signals or immunoregulatory cues may skew the ratio of M1/M2 macrophages and reverse the role of macrophages as accomplices in tumor progression to aides in tumor termination.^40, 45, 46^

Our findings showed a synergistic effect of CnB and IFN-*γ*, which resulted in a sudden burst of TRAIL expression in macrophages. We further investigated the synergistic tumor-suppressive effect of CnB and IFN-*γ in vitro* and *in vivo* and explored possible mechanisms of both the macrophage transformation and the interferon signaling amplification.

## Results

### CnB and IFN-*γ* act synergistically to enhance tumoricidal ability of RAW264.7 macrophages

**Figure 1:**
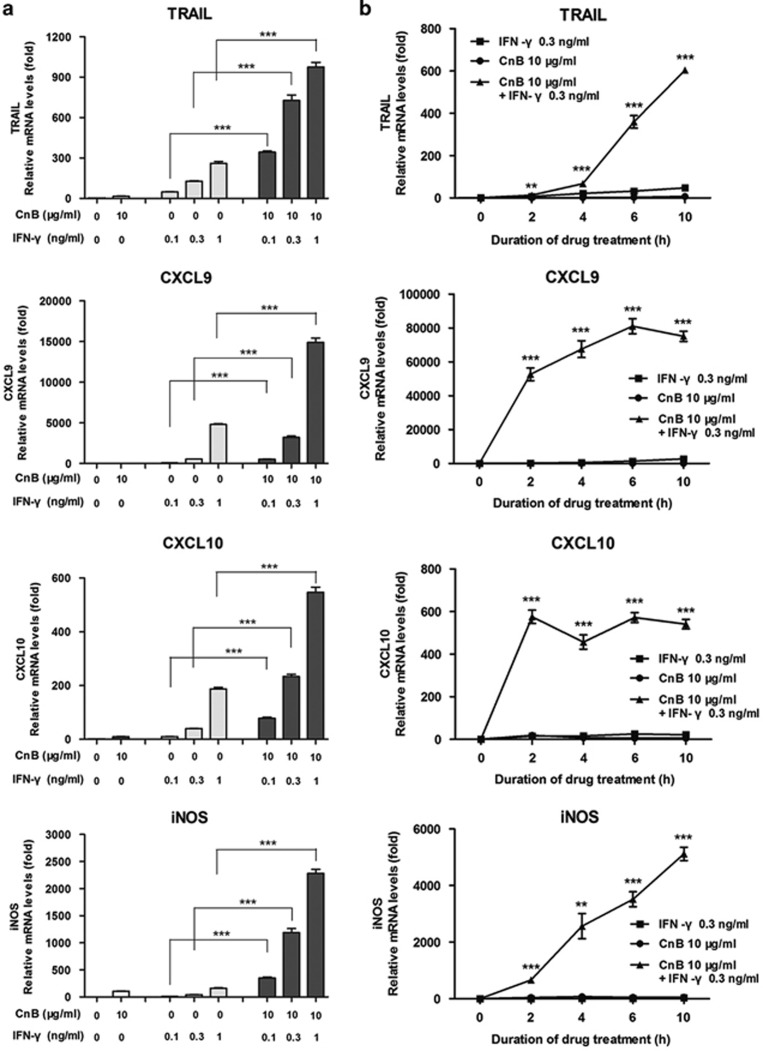

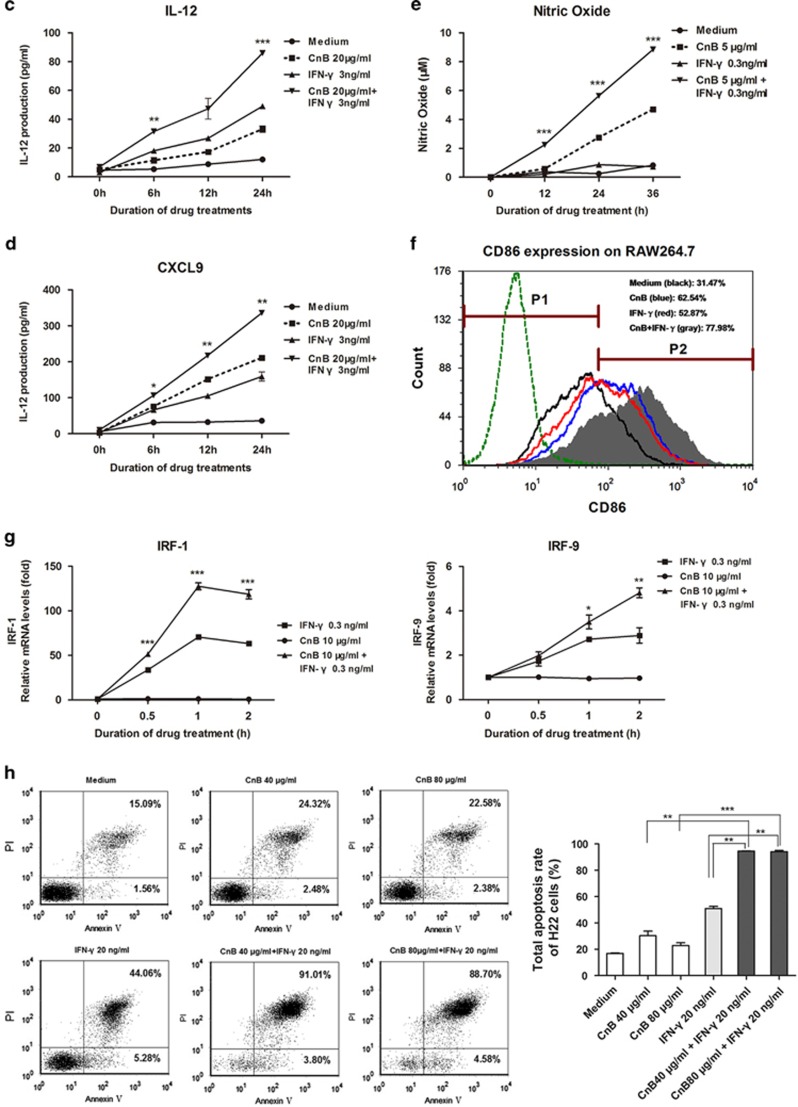
CnB and IFN-*γ* act synergistically in enhancing tumoricidal ability of RAW264.7 macrophages. (**a**) Quantitative PCR (qPCR) assay of the synergistic effect of CnB and IFN-*γ* in inducing TRAIL, iNOS, CXCL9, and CXCL10 transcripts (dose course). Cells were incubated with CnB (10 *μ*g/ml), IFN-*γ* (0.1–1 ng/ml), or CnB (10 *μ*g/ml)+IFN-*γ* (0.1–1 ng/ml) for 10 h. Results were normalized to *β*-actin expression and are presented as fold increases over the medium-only control. (**b**) qPCR assay of the synergistic effect of CnB and IFN-*γ* (time course). Cells were preincubated with CnB (10 *μ*g/ml) for 45 min; then the culture medium was removed and replaced with fresh medium with or without IFN-*γ* (0.3 ng/ml) and cultured for 2–10 h. (**c** and **d**) Enzyme-linked immunosorbent assay of the synergistic effect of CnB and IFN-*γ* (time course). **c**: IL-12; **d**: CXCL9. (**e**) The synergistic effect of CnB and IFN-*γ* in promoting nitric oxide release. (**f**) Flow cytometry analysis of CD86 expression. Cells were treated with CnB (20 *μ*g/ml), IFN-*γ* (3 ng/ml), or CnB (20 *μ*g/ml)+IFN-*γ* (3 ng/ml) for 24 h. P1, percentage of CD86-negative cells; P2, percentage of CD86-positive cells; Green: isotype control; Black: medium control; Blue: CnB; Red: IFN-*γ*; Gray: CnB+IFN-*γ*. (**g**) qPCR assay of the transcription factors IRF-1 and IRF-9 expression upon drug treatments (0–2h). (**h**) Synergistic antitumor effect of CnB and IFN-*γ in vitro*. Drugs-stimulated RAW264.7 cells were co-cultured with H22 hepatocarcinoma cells for 30 h. H22 cells in the supernatants were harvested, and apoptosis was analyzed by fluorescein isothiocyanate–Annexin V/propidium iodide staining. The graph to the right of panel (**h**) is a quantitative analysis of the total apoptosis rate of H22 cells. The total apoptosis rate was given by the percentage of Annexin V-positive cells. In panels **b**–**e** and **g**, the *t*-tests were carried out between CnB+IFN-*γ* treatment and IFN-*γ* treatment alone. *P<0.05, **P<0.01, ***P<0.001

As previously described, IFN-*γ* induces a robust transcriptional response in RAW264.7 macrophages^47^; this cytokine elicits the transcription upregulation of TRAIL, iNOS, CXCL9, and CXCL10 (Figure 1a). In contrast, CnB alone has minimal activity in inducing the activation of these genes (Figure 1a). Interestingly, the combination of CnB with IFN-*γ* elicites a robust synergistic increase in the mRNA levels in both a dose- (Figure 1a) and time-dependent manner (Figure 1b). The combination of CnB with IFN-*γ* also induces a significant synergistic increase in the protein levels (Figures 1c–f). CXCL9, CXCL10, and nitric oxide production are remarkably increased upon the combination treatment with CnB and IFN-*γ*, compared with the CnB or IFN-*γ* treatment alone (Figures 1c–e). CD86 expression on RAW264.7 is also further enhanced in response to the synergistic stimulation, compared with the single-drug treatments (Figure 1f). To understand how CnB can facilitate this synergism, we examined IRF-1 and IRF-9, two key transcription factors that control IFN-*γ*- mediated gene expression, including CXCL10, TRAIL, iNOS, and IFN-*β*.^48, 49, 50, 51^ In this study, we found that CnB and IFN-*γ* had a significant cooperative effect on the induction of these two critical transcription factors. Interestingly, CnB treatment alone did not induce IRF-1 and IRF-9 mRNA expression, but the transcript levels increased in response to IFN-*γ* (Figure 1g). These data suggest that CnB can modulate IFN-*γ*-dependent signaling in macrophages but do not address the impact of this synergy in the context of the immune antitumor response. In order to examine whether combination treatment could enhance the antitumor activity of immune cells, we co-cultured RAW264.7 macrophages with H22 hepatocarcinoma cells. Although treatment with CnB or IFN-*γ* alone only partially induced H22 cell apoptosis (Figure 1h, 30 and 50%, respectively), cotreatment of CnB with IFN-*γ*-activated RAW264.7 macrophages caused almost complete H22 cell apoptosis (Figure 1h, 95%).

### CnB synergizes with IFN-*γ* to polarize the mouse TAMs as well as human monocyte-derived macrophages to an M1-like differentiation

**Figure 2:**
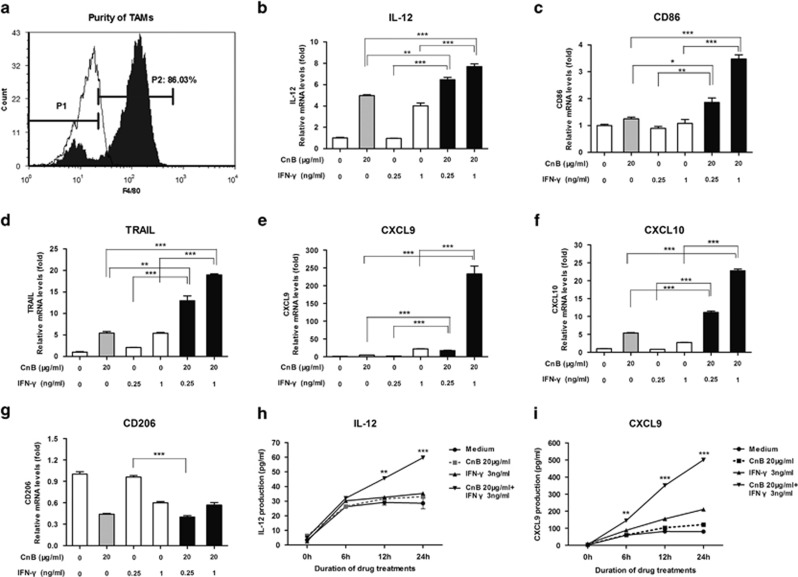
CnB synergizes with IFN-*γ* to polarize TAMs to M1-like phenotype *in vitro*. (**a**) Identification of TAMs' purity. P2 represents the percentage of F4/80-positive cells. Filled histogram represents the fluorescence of F4/80 antibody staining, and open histogram represents the fluorescence of isotype antibody staining. (**b**–**g**) Quantitative PCR analysis of M1 and M2 markers' expression in drugs-stimulated TAMs. Duration of drug treatments, 10 h. **b**: IL-12; **c**: CD86; **d**: TRAIL; **e**: CXCL9; **f**: CXCL10; **g**: CD206. (**h** and **i**) Enzyme-linked immunosorbent assay of IL-12 and CXCL9 production (time course). **h**: IL-12; **i**: CXCL9. In panels **h** and **i**, the *t*-tests were carried out between CnB+IFN-*γ* treatment and IFN-*γ* treatment alone. *P<0.05, **P<0.01, ***P<0.001

To determine whether the synergy between CnB and IFN-*γ* that mediates their antitumor activity was mediated through the conversion of macrophages from an M2-like phenotype to an M1-like phenotype, we isolated TAMs from B16-F10 melanoma from C57BL/6 mice. Flow cytometry analysis showed that the purity of these TAMs was >85% (Figure 2a). These cells were treated with CnB, IFN-*γ*, and CnB with IFN-*γ*. To determine whether CnB with IFN-*γ* could shift the phenotype of these cells, we examined several markers of the M1 (IL-12, CD86, TRAIL, CXCL9 and CXCL10) and M2 phenotype (CD206). Although CnB with IFN-*γ* strongly enhanced the transcript levels of the M1 phenotypic markers, IL-12, CD86, TRAIL, CXCL9 and CXCL10 (Figures 2b–f), CnB and IFN-*γ* did not synergize to downregule the M2-associated transcript CD206 (Figure 2g). CnB with IFN-*γ* also increased the protein levels of M1 phenotypic markers, such as IL-12 and CXCL9 (Figures 2h–i). These data suggest that CnB together with IFN-*γ* induce TAMs towards an M1-like phenotype.

**Figure 3:**
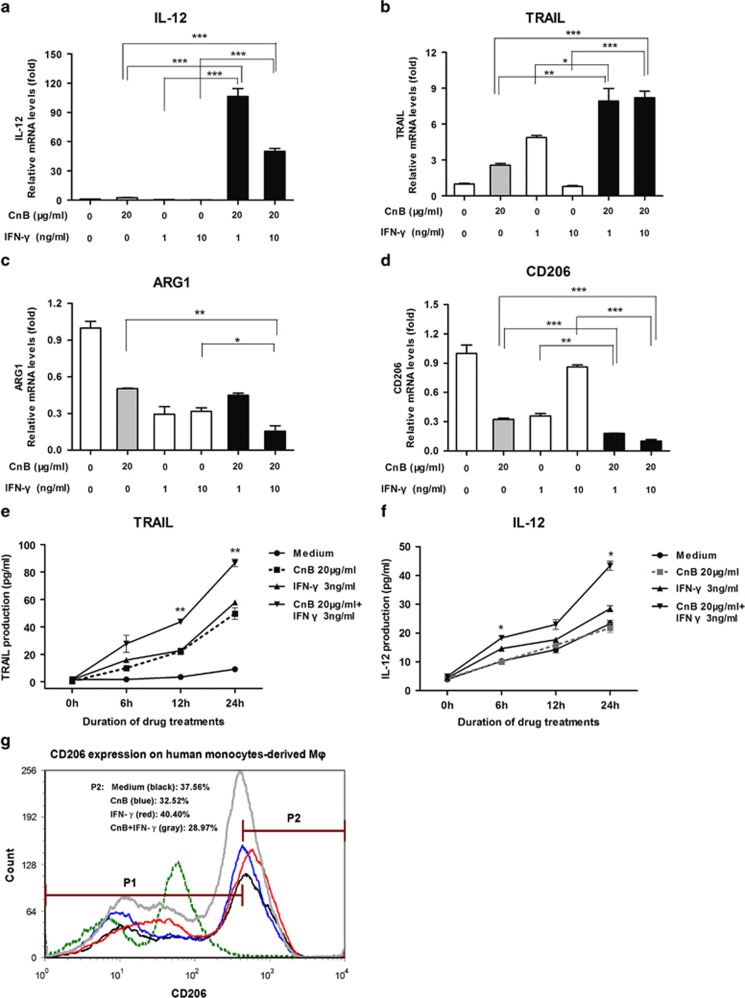
CnB synergizes with IFN-*γ* to skew human monocyte-derived macrophages to an M1-like phenotype. (**a**–**d**) Quantitative PCR assay of the synergistic effect of CnB and IFN-*γ* in inducing some M1 and M2 marker transcripts. **a**: IL-12; **b**: TRAIL; **c**: ARG1; **d**: CD206. (**e** and **f**) Enzyme-linked immunosorbent assay of TRAIL and IL-12 production upon CnB and IFN-*γ* stimulation. **e**: TRAIL; **f**: IL-12. (**g**) Flow cytometry analysis of CD206 expression on PBMC-derived macrophages. Cell were treated with CnB (20 *μ*g/ml), IFN-*γ* (3 ng/ml), or CnB (20 *μ*g/ml)+IFN-*γ* (3 ng/ml) for 24 h. P1, percentage of CD206-negative cells; P2, percentage of CD206-positive cells; Green: isotype control; Black: medium control; Blue: CnB; Red: IFN-*γ*; Gray: CnB+IFN-*γ*. In panels **e** and **f**, the *t*-tests were carried out between CnB+IFN-*γ* treatment and IFN-*γ* treatment alone. *P<0.05, **P<0.01, ***P<0.001

The data thus far examine the synergy of CnB and IFN-*γ* in mouse TAMs but do not address whether this phenomenon exists in human-derived cells. To examine whether CnB and IFN-*γ* can also shift human monocyte-derived macrophages toward an M1-like phenotype, we examined the levels of IL-12 in these cells. Although CnB and IFN-*γ* alone induced a mild increase of IL-12 mRNA levels (2.7-fold, CnB; 0.67-fold IFN-*γ*; Figure 3a) the combination of CnB with IFN-*γ* elicited a marked increase in this M1-associated transcript (107-fold; Figure 3a). Similarly, another M1 differentiation marker, TRAIL was also greatly upregulated in response to combination treatment (Figure 3b). Interestingly, in these human-derived cells, two M2-associated transcripts, CD206 and ARG1 expression, were cooperatively downregulated by CnB and IFN-*γ* combination treatment (Figures 3c and d). The synergy in the protein levels was not as robust as in the mRNAs levels, but we still can see a significant synergetic increase in the IL-12 and TRAIL production (Figures 3e and f) and a decrease in the CD206 expression (Figure 3g) on human monocyte-derived macrophages. These data suggest that the combination of CnB with IFN-*γ* can shift the macrophage profile towards an M1 phenotype in both mouse- and human-derived cells.

### CnB strongly induces phosphorylation of STAT1, and combined use of CnB and IFN-*γ* markedly enhances phosphorylation of STAT1 at Ser727 but not at Tyr701

**Figure 4:**
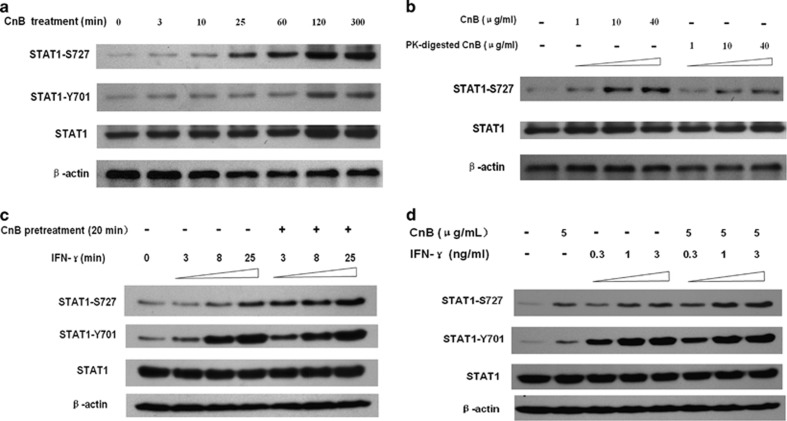
CnB stimulates phosphorylation of STAT1, and combination treatment with CnB and IFN-*γ* markedly enhances phosphorylation at Ser727 but not at Tyr701. (**a**) Western blotting detection of time-dependent phosphorylation of STAT1. RAW264.7 cells were treated with 20 *μ*g/ml CnB for 0-300 min. (**b**) Proteinase K-digested CnB loses the ability to induce phosphorylation of STAT1 at Ser727. Cells were incubated with CnB (1–40 *μ*g/ml) or proteinase K-digested CnB (1–40 *μ*g/ml) for 25 min. (**c**) CnB pretreatment enhances IFN-*γ*-induced phosphorylation of STAT1 at Ser727 but not at Tyr701 (time course). Cells were preincubated with or without CnB (20 *μ*g/ml) for 20 min, followed by incubation with IFN-*γ* (1 ng/ml) for 3–25 min. (**d**) Combined treatment with CnB and IFN-*γ* synergistically promotes STAT1 phosphorylation at Ser727 but not at Tyr701 (dose course). Duration of treatments, 25 min. Phosphorylation levels of STAT1 were quantified by densitometry and normalized to total STAT1 expression (Supplementary Figure S1)

To determine the molecular basis for the synergism of CnB and IFN-*γ*, we focused on STAT1, a key factor involved in the interferon signaling pathway, and tested whether CnB and IFN-*γ* synergized in promoting STAT1 phosphorylation. We examined CnB-induced phosphorylation of STAT1; CnB induced a robust and prolonged phosphorylation of STAT1 at Ser727 that lasted for 5 h after treatment (Figure 4a). Conversely, CnB induced weak and slow phosphorylation of STAT1 at Tyr701 (Figure 4a). Because CnB was expressed and purified from bacterial cultures, and LPS can also induce phosphorylation of STAT1,^52^ we sought to distinguish the CnB-induced STAT1 phosphorylation from the presumed LPS-induced STAT1 phosphorylation. In the proteinase K digestion experiment, we found that the proteinase K-digested CnB lost the ability to induce phosphorylation of STAT1 (Figure 4b), thus demonstrating that the CnB-induced STAT1 phosphorylation was not due to LPS contamination. We next studied the cooperative effect in a time-course analysis, showing that CnB pretreatment for 20 min significantly enhanced IFN-*γ*-induced STAT1 phosphorylation at Ser727 but not at Tyr701 (Figure 4c). Similar findings were obtained as a function of dose (Figure 4d). The time- and dosage-dependent results were consistent with the weak nature of the CnB induction of STAT1 phosphorylation at Tyr701.

### CnB-induced STAT1 phosphorylation is dependent on integrin *α*M-p38 pathway

**Figure 5:**
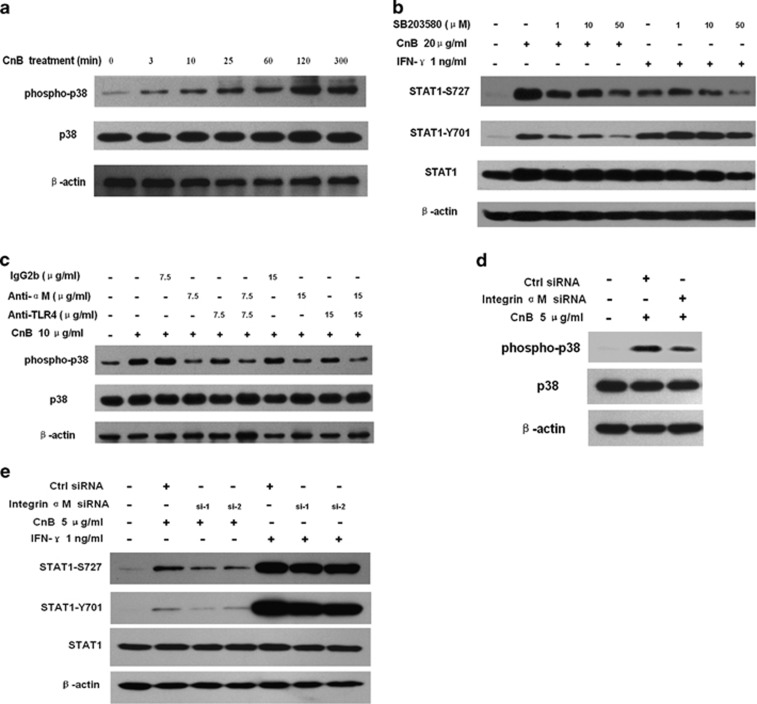
CnB-induced STAT1 phosphorylation is dependent on the integrin *α*M–p38 signaling pathway. (**a**) CnB promotes phosphorylation of p38 in a time-dependent manner. RAW264.7 macrophages were incubated with 20 *μ*g/ml CnB for 0–300 min, and then lysed for western blotting analysis. (**b**) The p38 inhibitor SB203580 markedly inhibits CnB-induced STAT1 Ser727 and Tyr701 phosphorylation. Cells were pretreated with SB203580 (1–50 *μ*M) for 30 min followed by CnB/IFN-*γ* treatment for another 30 min. (**c**) CnB-induced p38 phosphorylation is blocked by integrin *α*M antibody. Antibody concentrations, 7.5 or 15 *μ*g/ml; duration of antibody pretreatment, 45 min. (**d**) CnB-induced p38 phosphorylation is attenuated by integrin *α*M knockdown. Forty-eight hours after siRNAs transfection, cells were incubated with 5 *μ*g/ml CnB for 25 min. (**e**) CnB-induced STAT1 phosphorylation is attenuated by integrin *α*M knockdown. Forty-eight hours after siRNAs transfection, cells were incubated with CnB (5 *μ*g/ml) or IFN-*γ* (1 ng/ml) for 25 min. Phosphorylation levels of STAT1 and p38 were quantified by densitometry and normalized to total STAT1 or total p38 expression, respectively (Supplementary Figure S2)

Previous studies suggest that p38 MAPK is necessary for mediating the phosphorylation of STAT1 on Ser727.^31, 37, 38, 39^ Therefore, we investigated whether CnB activates p38 and whether CnB-induced STAT1 phosphorylation is also mediated by this kinase. CnB elicited a rapid p38 phosphorylation, which increased gradually over 300 min (Figure 5a). CnB-induced Ser727 phosphorylation and Tyr701 phosphorylation were both markedly (approximately 60–70%) inhibited by the p38 inhibitor, SB203580. In contrast, SB203580 could only inhibit IFN-*γ*-induced STAT1 Ser727 phosphorylation but not STAT1 Tyr701 phosphorylation (Figure 5b). These findings indicate that CnB-induced STAT1 Ser727 and Tyr701 phosphorylation are both mediated by p38 but IFN-*γ*-mediated signaling through STAT1 require additional regulatory components.

We have demonstrated that integrin *α*M is the specific receptor for CnB on macrophages.^18^ We therefore examined whether CnB-induced p38 phosphorylation was dependent on this receptor. Antibody blocking experiments showed that CnB-induced p38 phosphorylation was markedly inhibited by preincubation with an integrin *α*M-blocking antibody but only slightly affected by TLR4-blocking antibody. Combined preincubation with integrin *α*M and TLR4 antibodies did not significantly increase the inhibition (Figure 5c). Similarly, knockdown of siRNA knockdown of integrin *α*M dramatically inhibited CnB-induced p38 phosphorylation (Figure 5d). Consistent with these results, integrin *α*M knockdown also markedly attenuated CnB-induced STAT1 Ser727 and Tyr701 phosphorylation. As a control, IFN-*γ*-induced STAT1 Ser727 and Tyr701 phosphorylation was unaffected by integrin *α*M knockdown (Figure 5e). Collectively, these observations show that CnB-induced STAT1 phosphorylation is dependent on integrin *α*M-p38 signaling.

### CnB synergizes with IFN-*γ* to increase tumor-eradicating activity *in vivo*

**Figure 6:**
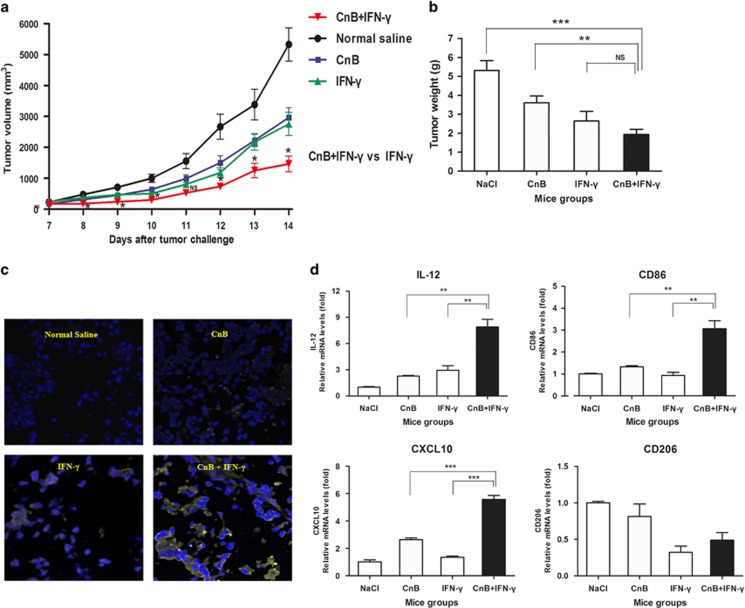
Synergistic antitumor effect of CnB and IFN-*γ* in a mouse melanoma primary tumor model. (**a**) Tumor volume curves for different drug treatment groups. Tumor volumes were measured every day from the seventh day after tumor challenge. Nine mice for each group. (**b**) Average tumor weight of each group. On the second day of the last injection, mice were killed and subjected to measurement of tumor weight. (**c**) Immunofluorescence detection of active caspase-3 in tumor slices by confocal microscopy. Six of mice in each group were subjected to tumor slice preparation. Yellow fluorescence represents caspase-3 expression; DAPI (4,6-diamidino-2-phenylindole; blue fluorescence) was used to stain the cell nuclei. Magnification, × 200. Picture above is a representative result of each group. (**d**) Quantitative PCR analysis of IL-12, CXCL9, CXCL10, and CD206 expression in TAMs. Data are representative result of three mice. *P<0.05, **P<0.01, ***P<0.001, NS: no significant differences

**Figure 7:**
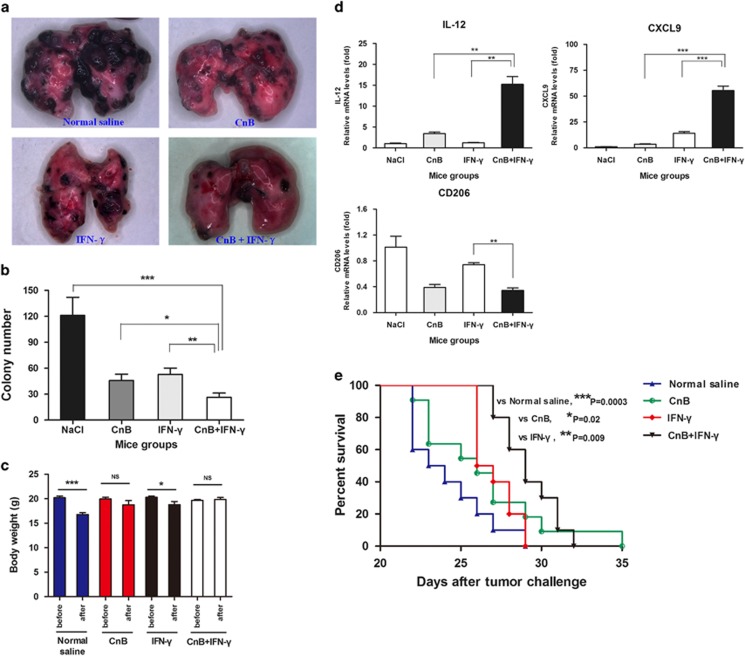
Synergistic tumoricidal acticity of CnB and IFN-*γ* in a mouse melanoma lung metastasis model. (**a**–**d**) Therapeutic effect of CnB and IFN-*γ*. Nine mice for each group. (**a**) Representative isolated lungs. (**b**) Mean melanoma colony numbers in the lungs per mouse. (**c**) Body weights before and after drug treatments. (**d**) Quantitative PCR analysis of IL-12, CXCL9, and CD206 genes' expression in TAMs. Data are a representative result of three mice. (**e**) Prophylactic effect of CnB and IFN-*γ*. Ten mice for each group. Survival following tumor challenge was recorded. Statistical differences between treatment groups were calculated by *t*-test (Graph **b**–**d**) and Gehan–Breslow–Wilcoxon test (Graph **e**). *P<0.05, **P<0.01, ***P<0.001, NS: no significant differences

Our data suggest that CnB primes IFN-*γ* signaling through enhancing STAT1 signaling in both mouse and human cell lines. To address whether this synergy can be utilized to enhance the macrophage antitumor activity *in vivo*, we used a melanoma tumor model as well as a melanoma lung metastasis model. In the primary melanoma tumor model, successive treatment with CnB or IFN-*γ* significantly prevented the melanoma growth. However, the combination of CnB with IFN-*γ* showed a stronger suppressive effect on the tumor volume and weight than either monotherapy groups (Figures 6a and b). Although the difference of tumor weight between the CnB with IFN-*γ* group and the IFN-*γ* alone group did not have a statistical significance, the average tumor weight of the CnB with IFN-*γ* group was lesser compared with the IFN-*γ* alone group. The timing of the mice killing may affect the statistical significance among the experimental groups. Furthermore, the combination treatment induced a marked increase in active caspase-3-positive cell in isolated tumors compared with CnB alone or IFN-*γ* alone (Figure 6c). Furthermore, IL-12, CXCL10, and CD86 genes transcription in TAMs from mice of the combination group was three or more folds higher than the single-drug treatment groups (Figure 6d); this implies a stronger M1-like phenotype transformation in TAMs by combination treatment. We next evaluated the cooperative effect of CnB and IFN-*γ* in a melanoma lung metastasis model, in which mice were injected with B16-F10 cells via the tail vein to achieve metastasis, showing that drug interventions either before or after B16 cells' inoculation can attenuate tumor metastasis and progression. In the therapeutic study, CnB and IFN-*γ* acted synergistically to reduce the colonization of melanoma cells to lung tissues (Figures 7a and b). As shown in Figure 7b, the average melanoma colony numbers in the combination group had been reduced to half to that of the CnB or IFN-*γ* group. In accordance to its desirable therapeutic efficacy, combined treatment effectively prevented mice body weight loss during tumor invasion (Figure 7c), which also implied a fact that combined treatment may not bring additional toxicity compared with single-drug treatments. In addition, two key cytokines, IL-12 and CXCL9 expression, in TAMs from mice of the combination group were many folds higher than in that of the single-drug treatment groups (Figure 7d), which was in agreement with the good performance in anatomy after combination treatment. In the prophylactic study, five successive combined treatments with CnB and IFN-*γ* before inoculation with B16-F10 melanoma cells significantly prolonged the survival of mice compared with treatment with normal saline (*P*<0.001), CnB alone (*P*<0.05), or IFN-*γ* alone (*P*<0.01) (Figure 7e). Taken together, these data indicate that CnB and IFN-*γ* have a marked synergistic antitumor effect *in vivo*.

## Discussion

Immunotherapy is often recommended as an adjuvant treatment to reduce the chance of cancer recurrence or metastasis. Interferons have been used clinically for treatment of renal cell carcinoma, melanoma, hairy cell leukemia, acute myeloid leukemia, glioma, and multiple myeloma. However, IFN-induced skin, neurological, endocrine, and immune toxicities have been a factor that restrict the amounts of interferon that can be used for effective cancer therapy.^53^ In order to reduce the toxicity from a single-drug treatment and bypass the issue of immune tolerance, IFNs are usually combined with other immunotherapeutic drugs, chemotherapy drugs, or molecular-targeted antitumor agents. For examples, combination treatment with PEG-interferon *α*-2b and recombinant interleukin-2 in phase I trial resulted in a 15% partial response, a 9.0 months median progression-free survival and a 31.9 months overall survival in patients with metastatic renal cell carcinoma. However, high-dose therapy may lead to grade 4 neutropenia and hypoxemia.^54^ In a phase I/II study, IFN-*γ* in combination with carboplatin and paclitaxel was applied to treat patients with advanced ovarian cancer and reached a 71% overall response rate. However, grade 3–4 neutropenia was observed in 74% of patients and other side effects, in particular peripheral neuropathies were also frequently observed.^55^ A phase III trial of bevacizumab plus IFN-*α versus* IFN-*α* monotherapy in patients with metastatic renal cell carcinoma showed that the median overall survival time was 18.3 months for bevacizumab plus IFN-*α* group and 17.4 months for the IFN-*α* monotherapy group, but there was significantly more grade 3–4 hypertension, anorexia, fatigue, and proteinuria for the bevacizumab plus IFN-*α* group.^56^

The examples above implicate that existing combinations between IFNs and other agents make some improvements on effectiveness compared with monotherapy, but these combination treatments are often accompanied with increased high-grade toxicity. Thus there is an urgent need for novel agents that can synergize with IFNs to achieve an antitumor activity with minimal side effects. Recombinant CnB protein is a newly reported candidate drug for tumor treatment by our laboratory. The advantages of CnB are not only in its ability to suppress tumor progression but also in its low toxicity *in vitro* and *in vivo*. Acute toxicity experiments indicated that mice can endure at least 50-fold the physiological dose of CnB.^20^ Successive injections of CnB at therapeutic doses for several months had no detrimental effect on mouse body weight and mouse liver and spleen indexes (data not shown). Thus CnB and IFN-*γ* combination treatment may provide a new way for overcoming the dosage limitation of IFN-*γ* and achieve effective antitumor activity with a low toxicity.

In the present study, we showed that CnB synergized with IFN-*γ* to transform human monocyte-derived macrophages and mouse TAMs into an M1-like phenotype. In fact, M1 and M2 macrophages often coexist in tumor microenvironment and the changed balance of M1 and M2 macrophages can influence tumor outcome.^44^ Targeting the phenotype transformation has been regarded as a novel strategy for cancer treatment. Rolny *et al.*^45^ reported that host-produced histidine-rich glycoprotein skewed TAM polarization away from the M2- to a tumor-inhibiting M1-like phenotype, thus inhibiting tumor growth and metastasis as well as tumor vessel abnormalization, which was also accompanied with improved chemotherapy. In another research by Beatty *et al.*,^46^ agonist CD40 mAb-activated macrophages rapidly infiltrated tumors and became tumoricidal by polarizing to a M1-differentiated phenotype and facilitated the depletion of tumor stroma, which did not necessarily depend on therapy-induced T cells. Besides CpG plus anti-interleukin-10 receptor antibody,^57^ GTP cyclohydrolase inhibitors,^58^ COX-2 inhibitors,^59^ microRNA-155,^60^ and Notch signaling agitator^61^ are also reported to be involved in TAM phenotype transformation. CnB plus IFN-*γ* greatly increased M1 marker (IL-12, CXCL9, CXCL10, CD86, etc.) expression in TAMs, whereas they selectively decreased some M2 markers (e.g., CD206) but not IL-10 expression. Moreover, combination of CnB and IFN-*γ* are able to transform TAMs into an M1-like differentiation; however, this transformation might be a bit different from the classic phenotype shift, which is represented with IL-12^high^ and IL-10^low^.

**Figure 8:**
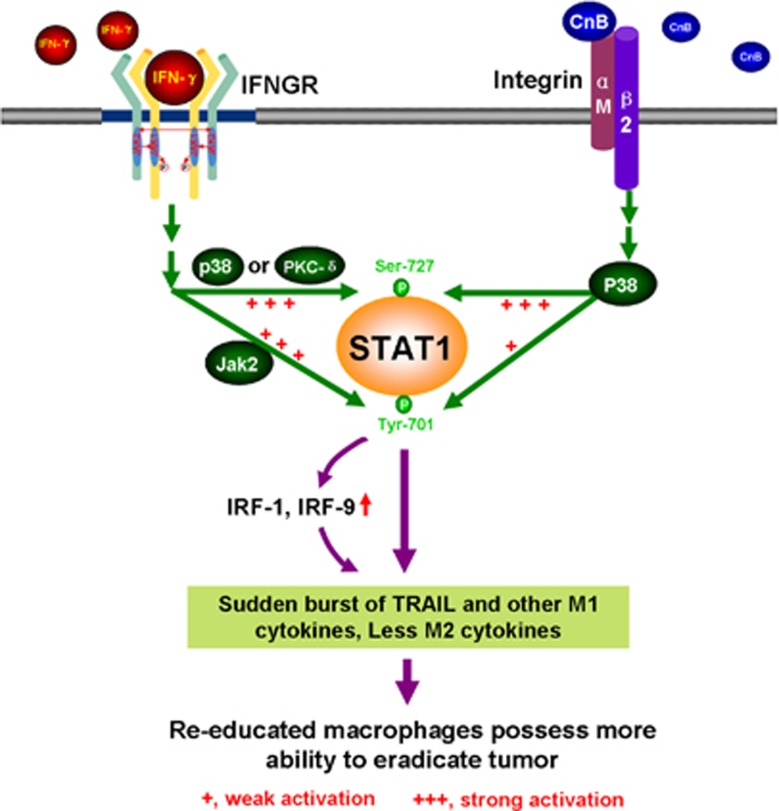
Diagram illustrating the mechanism of synergism between CnB and IFN-γ. CnB binds to an important macrophage membrane receptor integrin *α*M and activate p38 MAPK. Activated p38 promotes strong phosphorylation of STAT1 at Ser727 and weak phosphorylation at Tyr701. At the same time, IFN-*γ* binds to IFN-*γ* receptors (IFNGR) and strongly induces STAT1 Ser727 phosphorylation by p38 (or PKC-*δ*) and STAT1 Tyr701 phosphorylation by Jak2. Combination treatment with CnB and IFN-*γ* greatly enhances STAT1 serine and tyrosine phosphorylation and activates STAT1 maximally. The immense activation of STAT1 causes a sudden burst of TRAIL and other antitumor M1 cytokines directly or via the transcriptional factors IRF-1 and IRF-9 and finally render macrophages re-educated, possessing more ability to eradicate tumor. ‘+++' represents strong activation and ‘+' represents weak activation

We demonstrated in this study that CnB stimulated both STAT1 serine and tyrosine phosphorylation, but phosphorylation of the former was much more effective than that of the latter; this was different from the strong activation of STAT1 both at Tyr701 and Ser727 by IFN-*γ*. Further study showed that p38 was a critical modulator in CnB-initiated STAT1 serine and tyrosine phosphorylation, which was different from the pattern seen with IFN-*γ*, in which IFN-*γ*-induced STAT1 Tyr701 phosphorylation is thought to be mediated by Jak2, but IFN-*γ*-induced Ser727 phosphorylation is thought to be mediated by p38 MAPK or PKC-*δ*.^30, 31, 32^ Our data also supported that integrin *α*M, a newly reported receptor for CnB,^18^ is required for CnB-induced p38 activation and the subsequent STAT1 activation, consistent with an earlier report that engagement of *β*2-integrins can lead to p38 activation.^62^ To our knowledge, this is the first evidence that STAT1 phosphorylation can be mediated by integrin *α*M. To summarize, CnB and IFN-*γ* share different receptors, use different modulators, and represent different activation patterns, but they finally converge into a common key factor STAT1 and achieve a maximum activation, which leads to a rapid and intense amplification of IFN-*γ* signaling. Based on our findings and previous studies by other groups, we also proposed a model to summarize the basis of the synergism of CnB and IFN-*γ* (Figure 8).

The implications of the synergistic effect of CnB and IFN-*γ* are not limited to the area of pharmacology; this interaction may also have potential physiological importance. Because there is a high concentration of Cn in blood, amniotic fluid, and cytosol of cells,^63^ this could permit CnB to interact with monocyte-macrophages *in vivo*. As CnB is constitutively expressed in blood, and IFN-*γ* is stimulus dependent, we speculate that serum CnB may synergize with IFN-*γ* when hosts are invaded by microorganisms (i.e., viruses and bacteria) or malignant tumors, conditions in which IFN-*γ* levels would increase rapidly. This cooperation may rapidly induce extensive production of M1 cytokines that orchestrate a complex response to these various situations. The cooperation may actually have evolved into an immune surveillance mechanism that permits the host to respond more effectively and rapidly to internal and external dangers. This hypothesis should be further explored by neutralizing serum CnB with specific monoclonal antibodies.

## Materials and Methods

### Materials

Recombinant human CnB protein was prepared in our laboratory (The amino-acid sequences of human, mouse, and rat CnB protein are identical.). Endotoxin was removed with Cellufine ETclean S endotoxin-removing beads (Chisso Corporation, Tokyo, Japan). The CnB was >98% pure, and LPS contamination was <4 EU/mg. Proteinase K was from Roche Diagnostics (Indianapolis, IN, USA) and recombinant murine IFN-*γ* from Canspec Scientific Instruments Corporation (Shanghai, China). Phospho-STAT1 Ser727, phospho-STAT1 Tyr701, STAT1, phospho-p38, p38 antibodies, p38 inhibitor SB203580, recombinant human M-CSF, and recombinant human IFN-*γ* were from Cell Signaling Technology (Beverly, MA, USA). Antiactive caspase 3 antibody and Alexa Fluor 555-conjugated secondary antibody were from Abcam (Cambridge, UK). Alexa Fluor 488-conjugated F4/80 antibody and Alexa Fluor 488-conjugated rat IgG2b isotype control were from AbD serotec (Oxford, UK). Integrin *α*M-blocking antibody, TLR4-blocking antibody, and the rat IgG2b isotype control (functional grade purified) were purchased from eBioscience (San Diego, CA, USA). PE-labeled CD206 antibody, PE-labeled CD86 antibody, and related isotype antibodies were from BD PharMingen (San Diego, CA, USA). ELISA kits for TRAIL, IL-12, and CXCL9 were from R&D Systems (Minneapolis, MN, USA) and Uscn Life Science Inc. (Wuhan, China). Integrin *α*M siRNA was purchased from Ribobio Co., Ltd (Guangzhou, China), and the Annexin V/PI apoptosis detection kit was from Bender Med Systems (Vienna, Austria). Collagenase type I was from Invitrogen (Carlsbad, CA, USA). Ficoll was purchased from Amersham Biosciences (Uppsala, Sweden).

### Animals

C57BL/6 mice, 6–8 week of age, were purchased from Vital River Laboratories (Beijing, China). All animals were housed in microisolator cages, with autoclaved food and bedding to minimize exposure to viral and microbial pathogens, and all procedures were approved by the Institutional Animal Care and Use Committee.

### TAM purification

TAM purification procedure was derived and improved from Sierra *et al.*^64^ In brief, B16 melanoma tumors grown in C57BL/6 mice were surgically removed, minced, and left to disaggregate in 0.1% collagenase I-RPMI-1640 solution with constant stirring at 37 °C for 60 min. The suspension was filtered through a 200-mesh sieve to obtain single-cell suspension followed by treatment with red blood cell lysis buffer for 5 min. Cells were centrifuged and washed twice with serum-free medium and left to adhere in serum-free RPMI 1640 for 30 min. Nonadherent cells were washed away. More than 85% of the remaining adherent cells were TAMs, as assessed by macrophage-specific marker F4/80 using flow cytometry.

### Immunofluorescence

Immunofluorescent analyses were performed according to standard techniques. In brief, tumors were fixed in 4% formaldehyde in PBS and paraffin embedded. A 4-*μ*m thickness was cut from each paraffin block. After dewaxing and rehydration, the sections were microwaved for antigen retrieval in 10 mM citrate buffer for 10 min and then allowed to cool for 1 h at room temperature. Sections were blocked with 5% bovine serum for 20 min, followed by incubation with primary antibody (diluted 1 : 200) for 90 min, and then with secondary conjugated antibodies (diluted 1 : 400) for 20 min. Pictures were taken with a ZEISS LSM 700 microscope (Carl Zeiss, Jena, Germany).

### RNA extraction and real-time PCR

Total RNA was extracted from macrophages using an AxyPrep Multisource Total RNA Miniprep Kit (Axygen, Union City, CA, USA). Reverse transcription was performed with a PrimeScript 1st Strand cDNA Synthesis Kit (Takara, Kyoto, Japan). Real-time PCR primers were designed with the Primer 5.0 software (Premier Biosoft International, Palo Alto, CA, USA), and the sequences were as follows: mouse TRAIL forward 5′-GCCACAGACACTTTCGGTGTT-3′, reverse 5′-TGATCTCATTTTGCGGAAAGAA-3′ mouse CXCL9 forward 5′-TCCTTTTGGGCATCATCTTCC-3′, reverse 5′-TTTGTAGTGGATCGTGCCTCG-3′ mouse CXCL10 forward 5′-TCCTTGTCCTCCCTAGCTCA-3′, reverse 5′-ATAACCCCTTGGGAAGATGG-3′ mouse iNOS forward 5′-CAGATCCCGAAACGCTTCA-3′, reverse 5′-TGTTGAGGTCTAAAGGCTCCG-3′ mouse IRF-1 forward 5′-CAGAGGAAAGAGAGAAAGTCC-3′, reverse 5′-CACACGGTGACAGTGCTGG-3′ mouse IRF-9 forward 5′-CAGTCTAGGCTGTGCACCTG-3′, reverse 5′-TTCCTGGAGCATCAACTTCC-3′ mouse IL-12 forward 5′-GACCATCACTGTCAAAGAGTTTCTAGAT-3′, reverse 5′-AGGAAAGTCTTGTTTTTGAAATTTTTTAA-3′ mouse CD206 forward 5′-GCTTCCGTCACCCTGTATGC-3′, reverse 5′-TCATCCGTGGTTCCATAGACC-3′ mouse *β*-actin forward 5′-AGAGGGAAATCGTGCGTGAC-3′, reverse 5′-CAATAGTGATGACCTGGCCGT-3′ human IL-12 forward 5′-GCTATCTGAATGCTTCCTAA-3′, reverse 5′-AGTTCTTAATCCACATCCTATC-3′ human TRAIL forward 5′-AGCCTGGAATGGATTCGTGG-3′, reverse 5′-GTGGCGGTTTTTGTCCTTCA-3′ human CD206 forward 5′-CTACTATGTCTTGGAATGATAT-3′, reverse 5′-TAACTGGTGGATTGTCTT-3′ human ARG1 forward 5′-TTAGATATAATGGAAGTGAA-3′, reverse 5′-GTTAAGGTAGTCAATAGG-3′ and human *β*-actin forward 5′-GTGACAGCAGTCGGTTGGAG-3′, reverse 5′-AGTGGGGTGGCTTTTAGGAT-3′. PCR was performed on an Applied Biosystems 7500 Real Time PCR system using SYBR Green Master Mix reagent (Applied Biosystems, Foster City, CA, USA). Reactions were performed in triplicate or duplicate.

### Western blotting analysis

Western blot analysis was performed on whole cell lysates of RAW264.7 macrophages. They were resolved by SDS-PAGE under reducing conditions and electrotransferred to a PVDF membrane (Millipore, Bedford, MA, USA). The membrane was blocked with 5% nonfat milk and incubated with primary antibody overnight at 4°C, followed by incubation with HRP-conjugated goat-anti-rabbit IgG for 1 h at room temperature. Protein bands were detected with SuperSignal ECL reagents (Pierce, Rockford, IL, USA) and visualized by autoradiography.

### RNAi

RAW264.7 macrophages were transfected with siRNAs targeting mouse integrin *α*M or control siRNA using HiPerFect Transfection Reagent (Qiagen, Valencia, CA, USA) according to the manufacturer's instructions. In brief, cells were seeded at 3 × 10^5^ cells per well in 12-well plates and incubated for 6 h in the presence of 750 ng siRNA complexed with 18 *μ*l HiPerFect. The transfected cells were used after 48 h. The sequences of the siRNAs targeting integrin *α*M were as follows: siRNA1: sense 5′-GCACUGAGAUCCUGUUUAA dTdT-3′, antisense 3′-dTdT CGUGACUCUAGGACAAAUU-5′ and siRNA2: sense 5′-GGAGAAUACUUAUGUGAAU dTdT-3′, antisense 3′-dTdT CCUCUUAUGAAUACACUUA-5′.

### Flow cytometry

In the H22 cells and RAW264.7 cells co-culture experiment, apoptosis of the H22 cells was examined by Annexin V–FITC/PI staining. The H22 cells were washed twice with ice-cold PBS and stained with 10 *μ*l Annexin V and 5 *μ*l PI in 200 *μ*l binding buffer for 15 min at room temperature. After staining, 300 *μ*l of binding buffer was added to each tube, and samples were analyzed with a FACSVantage SE (BD Biosciences, San Jose, CA, USA). Unstained cell samples and cells stained with Annexin V or PI only were prepared for fluorescence compensation. In the F4/80, CD86, and CD206 expression assay, adherent TAMs or human monocyte-derived macrophages were collected by gentle scraping using the PBS/BSA buffer and washed twice with the same buffer, and then cells were incubated with flow cytometry antibodies (5 *μ*g/ml) or isotype antibody (5 *μ*g/ml) on ice for 20–30 min. After staining, cells were washed twice and subjected to flow cytometry analysis.

### Preparation of human monocyte-derived macrophages

Blood from healthy donors was collected in heparin-treated tubes and peripheral blood mononuclear cells (PBMCs) were isolated by standard Ficoll density-gradient centrifugation. PBMCs were plated in 12-well plates (1.6 × 10^6^ cells/well), and after 2 h of incubation for adherence, the medium was replaced with RPMI-1640 containing 50 ng/ml M-CSF. Adhered cells were incubated at 37 °C in 5% CO_2_ for 4–5 days to induce differentiation into macrophages, with a change of half of medium at day 3.

### Murine melanoma models

#### Mouse melanoma primary tumor model

C57BL/6 mice were inoculated at right armpit with 1 × 10^6^ B16-F10 melanoma cell and divided into four groups (normal saline group, CnB group, IFN-*γ* group, CnB+IFN-*γ* group; nine mice for each group). Three days after inoculation, mice were given 10 successive injections (i.p., 1 injection/day) of normal saline, CnB (20 mg/kg/day), IFN-*γ* (50 *μ*g/kg/day) and CnB plus IFN-*γ* (20 mg CnB+50 *μ*g IFN-*γ* /kg/day), respectively. Tumor volumes were measured every day from the seventh day after tumor challenge. On the second day of last injection, mice were killd and subjected to measurement of tumor weight; then six of mice in each group were subjected to tumor slice preparation and others were used for separating TAMs.

#### Mouse melanoma lung metastasis model

In a therapeutic study, C57BL/6 mice were randomly divided into four groups (nine mice for each group) and injected i.v. with 3 × 10^5^ B16-F10 cells via the tail vein. From the second day following inoculation, mice were injected i.p. with normal saline, CnB (20 mg/kg), IFN-*γ* (50 *μ*g/kg) or CnB (20 mg/kg) plus IFN-*γ* (50 *μ*g/kg) every day for 18 days. On the second day after the last administration, mice were killed, and the lungs were removed for photograph, colony counting, and macrophages isolation. In a prophylactic study, C57BL/6 mice were randomly divided into four groups (10 mice for each group) and injected i.p. with normal saline, CnB (5.0 mg/kg), IFN-*γ* (50 *μ*g/kg) and CnB (5.0 mg/kg) plus IFN-*γ* (50 *μ*g/kg), respectively, every 2 days for 10 days; then the mice were injected i.v. with 3 × 10^5^ B16-F10 cells in 200 *μ*l normal saline via their tail vein. Survival following tumor challenges was recorded.

### Statistical analysis

Data were expressed as means and S.E.Ms. Data were analyzed by two-tailed *t*-tests and Gehan–Breslow–Wilcoxon test (for survival curve analysis). *P*<0.05 was considered statistically significant.

## Abbreviations

CnB: calcineurin B subunit
STAT1: signal transducer and activator of transcription 1
Cn: calcineurin
TRAIL: TNF-related apoptosis-inducing ligand
Jak2: Janus kinase 2
MAPK: mitogen-activated protein kinase
PKC-*δ*: protein kinase C-*δ*
IRF: interferon regulatory factor
CXCL: CXC-chemokine ligand
iNOS: inducible nitric oxide synthase
ARG1: arginase-1
TAM: tumor-associated macrophage
PBMC: peripheral blood mononuclear cell
