# Supplementary material for: The synergistic interaction between the calcineurin B subunit and IFN-γ enhances macrophage antitumor activity
Source: Cell Death Dis. 2015 May 7;6(5):e1740–. doi: 10.1038/cddis.2015.92 (PMC4669720; doi:10.1038/cddis.2015.92)
Supplement: Supplementary Figures [file cddis201592x1.doc]

**
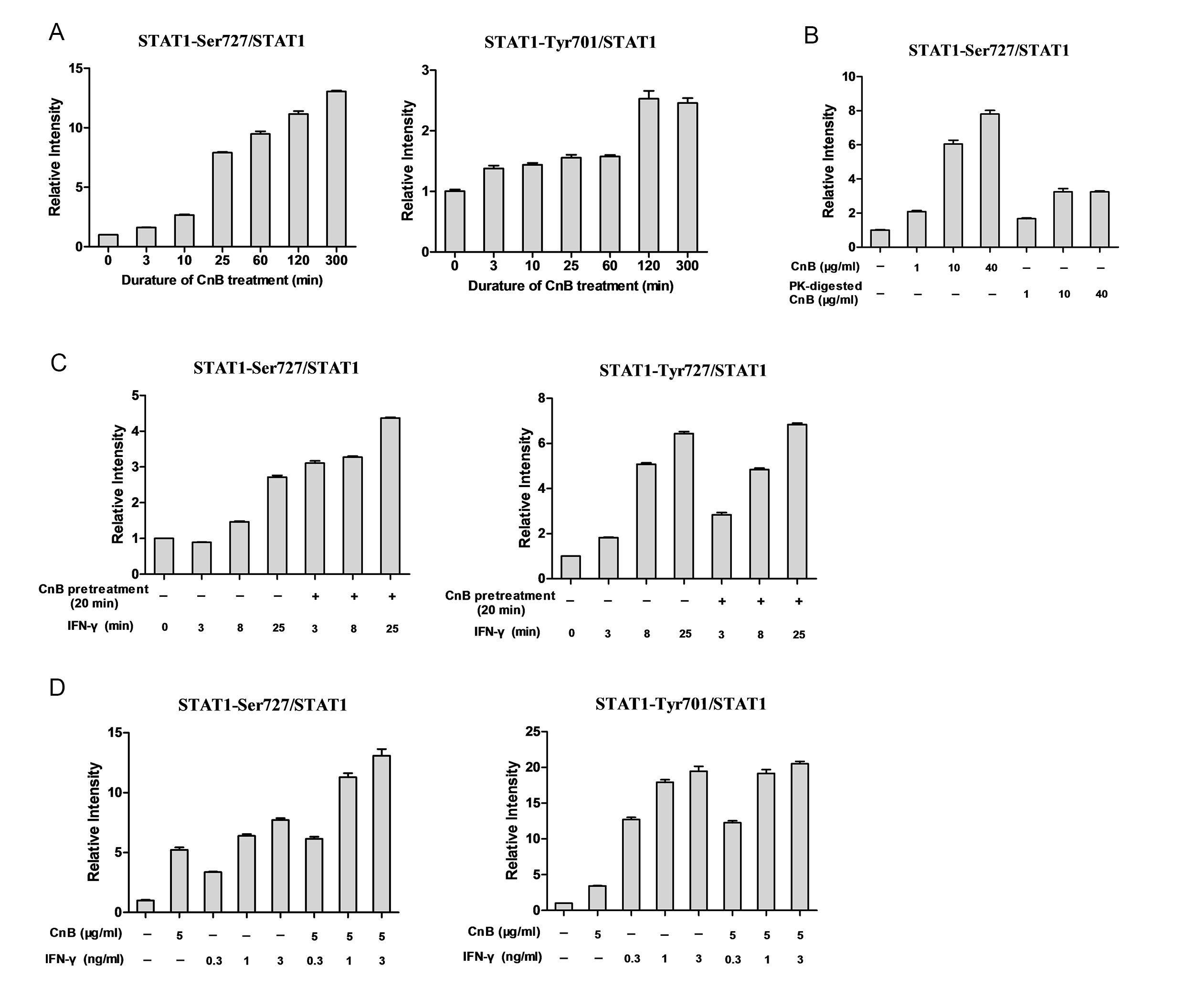
**

**Figure S1** **Densitometry analysis of phosphorylation levels of STAT1 in Figure 4**

Phosphorylation of STAT1 at Ser727 or Tyr701 are quantified by densitometry using Image J software and normalized to the total STAT1 expression. The graphs “A, B, C, D” in Figure S1 are quantitative analyses of graphs “A, B, C, D” in Figure 4, respectively.

**
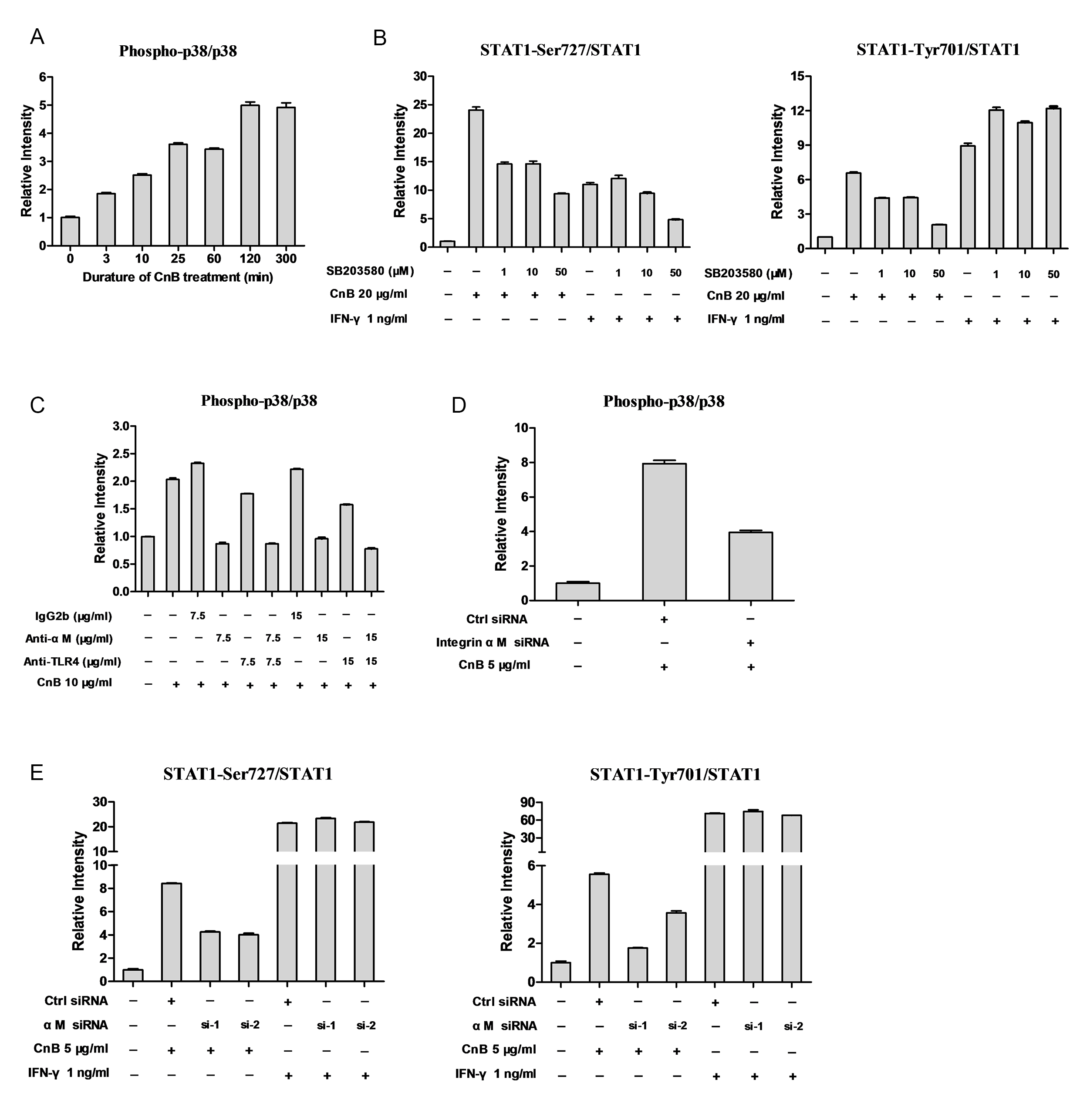
**

**Figure S2 Densitometry analysis of phosphorylation levels of STAT1 and p38 in Figure 5.**

Phosphorylation of STAT1 at S727 and Tyr701 and phosphorylation of p38 were quantified by densitometry using Image J software and normalized to total STAT1 or total p38 expression. The graphs “A, B, C, D, E” in Figure S2 are quantitative analyses of graphs “A, B, C, D, E” in Figure 5, respectively.
